# The impact of COVID-19 lockdown on physical activity and sedentary behaviour in secondary school teachers: a prospective cohort study

**DOI:** 10.1186/s12889-024-18954-4

**Published:** 2024-06-05

**Authors:** Yanni Verhavert, Tom Deliens, Lara Stas, Elke Van Hoof, Benedicte Deforche, Dirk Aerenhouts, Peter Clarys, Evert Zinzen, Kristine De Martelaer

**Affiliations:** 1 Department of Movement and Sport Sciences, Vrije Universiteit Brussel, Pleinlaan 2, Brussels, 1050 Belgium; 2 Core Facility - Support for Quantitative and Qualitative Research (SQUARE), Vrije Universiteit Brussel, Pleinlaan 2, Brussels, 1050 Belgium; 3 Department of Biostatistics and Medical Informatics, Vrije Universiteit Brussel, Laarbeeklaan 101, Brussels, 1090 Belgium; 4 Brussels, Belgium; 5 Department of Public Health and Primary Care, Ghent University, C. Heymanslaan 10, Ghent, 9000 Belgium

**Keywords:** Confinement, Quarantine, Energy expenditure, Longitudinal, Pandemic, Teachers, Healthy lifestyle

## Abstract

**Background:**

Mid-March 2020, Belgium went in lockdown to combat the COVID-19-pandemic. Having to provide school-based day care and adapt to online teaching, while all social, cultural and sports events and activities were cancelled, secondary school teachers’ physical activity (PA) and sedentary behaviour (SB) may have been affected considerably. This study investigates the impact of the first Belgian lockdown on PA and SB in Flemish secondary school teachers.

**Methods:**

This prospective cohort study was conducted throughout the 2019–2020 school year. PA and SB measured in March/April 2020 were compared with a pre-lockdown measurement in January/February 2020. Other pre-lockdown measurements (September/October 2019 and November/December 2019) and one other during-lockdown measurement (May/June 2020) allowed us to control for confounding. Validated questionnaires were used to assess participants’ PA and SB. Generalized linear mixed models were applied in R.

**Results:**

Among 624 participants (77·2% females, 43·3 ± 10·3 years), increases were observed for total PA (+ 108 min/week; *p* = 0·047), moderate PA (+ 217 min/week; *p* = 0·001), domestic and garden PA (+ 308 min/week; *p* < 0·0001) and leisure-time PA (+ 131 min/week; *p* < 0·0001), whereas work-related PA (-289 min/week; *p* < 0·0001) and active transportation (-38 min/week; *p* =0·005) decreased. No differences were observed for walking (*p* = 1·0) and vigorous PA (*p* = 0·570). Increases were found for total SB (+ 972 min/week; *p* < 0·0001), work-related SB (+ 662 min/week; *p* < 0·0001) and leisure-time SB (+ 592 min/week; *p* = 0·0004), whereas transport-related SB (-290 min/week; *p* < 0·0001) decreased.

**Conclusion:**

During the lockdown, we found in our sample that Flemish secondary school teachers showed an increase in SB that was 9 times as high as their PA increase. As a government, education network or school, it is crucial to sensitize, promote, and facilitate sufficient MVPA and/or walking, but likewise to discourage SB during pandemic-induced lockdowns.

**Supplementary Information:**

The online version contains supplementary material available at 10.1186/s12889-024-18954-4.

## Introduction

On March 13, 2020, the Belgian Federal Government installed a first set of rigorous measures to combat the COVID-19 pandemic, followed by a tightening of these measures on March 18. This first COVID-19-induced lockdown included several measures impacting our daily lives dramatically. Schools cancelled all classes at first and then, in analogy with the universities, changed to digitalized long-distance learning, bars and restaurants were closed down, and all social, cultural and sports events and activities were cancelled. This included organised sports and group-based physical activities. Also, non-essential workers were asked to work from home, and for a major part of them, these measures even resulted in being technically unemployed.

These drastic changes in work and lifestyle are most likely to impact people’s physical activity (PA) and sedentary behaviour (SB). Low PA and high SB levels show a clear association with both acute and non-acute health problems [1, 2]. Inactive and sedentary people show greater risk of developing overweight and obesity, and other non-communicable diseases, such as cardiovascular disease, type 2 diabetes, hypertension, and cancer [1], but also have an apparent link with severe COVID-19 illness [3]. It is also known that exercise positively affects the immune system, which may be crucial in fighting off bacterial or viral infections [4]. Besides physical health, inadequate PA and excessive SB are associated with poor mental health, such as depression, anxiety and burn-out [5–8]. The above illustrates the importance of an active lifestyle, especially during a virus outbreak and subsequent lockdown circumstances.

Secondary school teachers have been affected considerably by the aforementioned lockdown measures as, in Belgium, they had to provide school-based day care at the very beginning of the lockdown (from March 16 to April 3, 2020; i.e., the start of the Easter vacation) and adapt to digitalised long-distance learning (from April 20, 2020 until May 18, 2020), and even hybrid teaching during the remainder of the school year (until June 30, 2020). In addition, when teachers had to return to the classroom, they had to deal with students who needed to recover (academically and emotionally) from the strict pandemic measures and they were putting their own health, and that of their families, at risk as the pandemic was still ongoing [9, 10]. Research also found that teachers reported higher anxiety levels than other professions and that remote teachers experienced higher levels of distress compared to teachers teaching in person [11]. As PA and SB are found to be associated with mental health [12–17], and as teachers’ mental health was found to be impacted by the pandemic, it is essential to investigate the impact of the pandemic on lifestyle factors such as PA and SB within this specific population. As this study is part of a larger longitudinal study on burnout and lifestyle among secondary school teachers, the present study was only able to focus on secondary school teachers. While research in this specific population is lacking, we hypothesise that the lockdown measures have had a significant impact on secondary school teachers’ lifestyle, including PA and SB.

A recent systematic review reported lockdown-induced decreases in PA and increases in SB across several countries and populations (excluding secondary school teachers) [18]. In Belgium, however, increases in both PA and SB were found in a general adult population [19]. Unfortunately, no details on the duration of PA and SB activities, PA intensity or the contextual domains in which PA and SB appeared, were provided [19]. However, a few non-Belgian studies focused on different PA domains, showing that the impact of COVID-19 on PA was domain-specific (i.e., decrease in sport levels and active travel, increase in housework/gardening and habitual PA, and no difference for light outdoor activities during the first lockdown) [20, 21]. Also, due to the acute nature of the corona-crisis, many studies that have been published (including the aforementioned Belgian study), investigated the effect of the lockdown measures on PA and/or SB using a cross-sectional and retrospective approach [18]. Those who did use a longitudinal study design most often included only two measurement points [22]. As such research designs are prone to considerable bias, prospective research with multiple measurement points is needed to confirm these findings. Also, due to the different types of lockdown measures per country, generalisability of other countries’ findings is limited. Hence, the present prospective cohort study aimed to add to the limited knowledge resulting from the above described and sole Belgian study [19].

The present study is part of a larger longitudinal follow-up study (launched in 2019) and therefore presents unique natural experiment data on how PA (including duration, intensity and contextual domain) and SB (including duration and contextual domain) in Flemish secondary school teachers have been impacted by the first COVID-19 lockdown.

## Methods

### Participants

A non-probability cluster sampling strategy was used to recruit Flemish secondary school teachers. In August and September 2019, all secondary schools in Flanders (Belgium) were contacted through e-mail and telephone. To increase the response and participation rate, the Flemish Department of Education (*Vlaams Departement Onderwijs*) as well as all education networks (i.e., Flemish community schools, subsidised public schools, subsidised free schools) were involved in the recruitment and were asked to promote the study among all school principals. To stimulate school involvement, a convenient selection of schools in Flanders were visited to promote our study face-to-face. Schools that were willing to participate in the study were asked to send an e-mail with a link to an online questionnaire to their entire teaching staff. Furthermore, the same link was spread through social media (e.g., Facebook, Twitter) and by posting advertisements via the Flemish Department of Education. Teachers being in sick leave or having a distorted physical activity/dietary pattern (by e.g., injuries, diseases, following a diet) were excluded from the final sample. As this study was part of a larger longitudinal follow-up study, in which we questioned distorted physical activity/dietary patterns by one question, we could not differentiate between the two. As a result, participants who reported being distorted in either category were excluded.

### Design and procedure

**Fig. 1:**
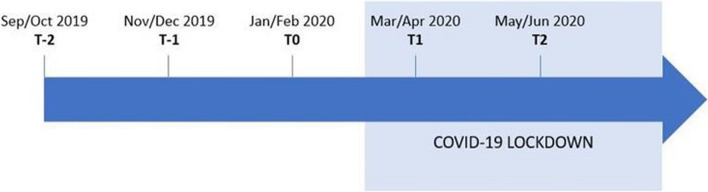
Timing of the measurements

This prospective cohort study is part of a larger longitudinal study, including six measurements throughout the 2019–2020 school year, i.e., Sep/Oct, Nov/Dec, Jan/Feb, Mar/Apr, May/Jun, and Jul/Aug. For the purpose of the present study (i.e., measuring the impact of COVID-19 lockdown on secondary school teachers’ PA and SB), the Jan/Feb measurement (Jan 27 – Feb 11, 2020) will serve as baseline (T0). The measurement performed in Mar/Apr (Mar 23 – Apr 7, 2020), which is five days after the installation of the lockdown measures, will serve as measurement under lockdown-exposure (i.e., primary endpoint (T1)). The measurements prior to T0 (i.e., Sep/Oct (T-2) and Nov/Dec (T-1)) will serve as ‘pre’ control measurements, whereas the measurement after T1 (i.e., May/Jun (T2)) will serve as ‘post’ control measurement. The Jul/Aug measurement was omitted due to anticipated summer holiday bias. The timeline of the measurements is displayed in Fig. 1.

At each time point participants were asked to complete an online questionnaire, including sample characteristics and primary outcome measures. Sample characteristics include socio-demographics, work-related information, and other health-related variables. Primary outcomes in the present study are PA and SB. During each measurement period of two weeks, three reminders were sent to the non-responders, each on the fourth, eighth and eleventh day after activation of the online questionnaire.

### Sample characteristics

Socio-demographics include sex, age, highest diploma (i.e., secondary school degree, post-secondary school degree or certificate, Bachelor’s degree, Master’s degree, PhD degree), having an extra job (yes/no), marital status (i.e., single, married, unmarried, living together with partner, divorced, widowed), having children (yes/no) and ethnicity (i.e., White – European, White – other, North-African, Afro-American, Indian, Middle-Eastern, South-Asian, Southeast-Asian, other). Work-related factors include education network (i.e., Flemish community schools, subsidised free schools, subsidised public schools) and total working hours per week. Health-related variables include self-reported height and weight (from which body mass index (BMI; kg/m²) was calculated) and smoking status (yes/no).

### Primary outcome measures

#### Physical activity

The validated International Physical Activity Questionnaire (IPAQ – Dutch long version) was used to estimate PA domains and intensities during the last seven days [23]. This self-report questionnaire includes 31 items and assesses four contextual PA domains: [1] work-related [2], transport-related [3], domestic and garden, and [4] leisure-time PA. The participants were asked to fill in the number of days and the amount of time (hours and minutes) spent in three different PA intensity levels within each domain, namely [1] walking [2], moderate-intensity PA, such as carrying light loads, washing windows, cycling or swimming at a regular pace, and [3] vigorous-intensity PA, such as heavy lifting, aerobics, running and fast cycling or fast swimming (as specified by the IPAQ). The outcome measures are domain- and intensity-specific PA as well as total PA expressed in min/week. Multiple criteria from the IPAQ scoring protocol were applied [24]: [1] only values of ten or more minutes of activity were retained; [2] non-relevant observations were excluded (e.g., answering in step counts instead of minutes); [3] PA levels higher than 960 min/day (i.e., 16 h/day) were excluded, as this would be unrealistic. Total scores per domain were calculated by multiplying the frequency of each PA per week by its duration expressed in minutes. Next, the domains were combined into total walking, moderate-intensity PA, and vigorous-intensity PA. Lastly, total PA was calculated by summing all items. It should be mentioned that total light-intensity PA, in which walking is just one component, is not questioned in the IPAQ. Therefore, total PA in this study only represents walking and moderate-to-vigorous-intensity PA. Note that the IPAQ scoring protocol includes a section “Truncation of Data Rules”, which is not applied in the current study. The protocol states that “this rule attempts to normalize the distribution of levels of activity which are usually skewed in national or large population data sets” [24]. Instead of truncating and forcing data into a normal distribution, we opted to tailor the statistical analyses to the non-normal data distributions (see Statistical analysis section). The IPAQ has fair to good psychometric properties (reliability: ρ = 0.80 and validity: *r* = 0.30) [25].

#### Sedentary behaviour

SB was assessed by using the Dutch version of the validated context-specific sedentary behaviour questionnaire for adults developed by Busschaert and colleagues [26]. This self-report questionnaire assesses SB in three domains: [1] work-related [2], transport-related, and [3] leisure-time SB. Participants were asked to specify how much time they spent sitting/lying down during the last seven days (weekdays and weekend days separately) within each domain. The outcome measures are domain-specific SB as well as total SB expressed in min/week (i.e., sum score of minutes during the week and weekend). Participants were asked to fill in the number of days and the amount of time spent sitting/lying for several items/activities (e.g., TV watching, computer use, reading) within each of the three domains. For each item, a specific time interval could be chosen; e.g., 1 to 15 min, 15 to 30 min, 30 to 60 min, 1 to 2 h, etc. Midpoint values (e.g., 7.5 min, 22.5 min, 45 min, 90 min, etc.) of each test item interval were calculated. As it was not mentioned in the protocol how the upper limit time intervals “more than seven hours a day” and “more than eight hours a day” had to be interpreted, it was decided to consider these time intervals as 450 min and 510 min, respectively. Total sedentary time for an average day was estimated by summing all midpoint values of the specific SB contexts (weekdays and weekend days separately) and was estimated as follows: ((total sedentary time on a weekday * 5) + (total sedentary time on a weekend day * 2))/7. Although not explicitly mentioned in the paper of Busschaert and colleagues [26], but consistent with the IPAQ protocol, we decided to exclude participants with SB levels higher than 960 min/day (i.e., 16 h/day) from the analysis.

### Patient and public involvement statement

Patient and public involvement was not appropriate for this study.

### Equity, diversity, and inclusion statement

Secondary school teachers from multiple geographical regions, urban and rural communities and different education networks were recruited for this study. Participants could report their sex, diploma and ethnicity. The author team included early, middle and late career researchers with balance from people who identify as male and female.

### Statistical analyses

All data were analysed using R (R core Team, 2019; R Studio version 3.6.2) and SPSS (version 27). P-values < 0.05 were considered statistically significant, whereas p-values between 0.05 and 0.10 were considered marginally significant. Representativeness of the sample at baseline (T0) was assessed by conducting two proportions z-tests. Drop-out analyses between baseline (T0) and the primary endpoint (T1) were conducted to assess possible selection bias of the retention group. In the first analysis, participants of whom we had data at T0 and T1 (i.e., retention group) were compared to participants of whom we only had data at T0 (i.e., drop-out group). As the generalized mixed models that we used typically include all available observation points, we decided to perform a second analysis in which we compared participants of whom we had data at T0 and T1 to participants of whom we only had data at T1. Independent samples *t*-tests, Mann-Whitney U tests and chi² tests were conducted to detect possible differences between the drop-out group and retention group regarding total PA, total SB, sex, age, ethnicity, marital status, having children, smoking status, diploma, having an extra job, education network and BMI.

Multilevel models were used for data analysis. Preliminary analyses checked if a three level model was advised (repeated measures clustered within participants, participants clustered within schools) using graphical representations and by inspecting the amount of variance explained by each cluster. If necessary, one (or both) levels were dropped. Possible confounders, such as age and sex, were checked, but seemed to have no significant effects, and therefore no adjustments were made in the statistical models. The PA scale scores were non-normally distributed with continuous, positively skewed non-negative values. The SB scales also contained non-negative continuous values, but with less severe skewness. For both outcome variables, Gamma and Gaussian generalized linear mixed models were constructed using the R package lme4 [27]. To decide upon the model (i.e., Gamma or Gaussian) and link functions (i.e., log, inverse or identity), Bayesian Information Criterion (BIC) values were compared and a likelihood ratio test was performed (lrtest() function of the R package lmtest [28]). The model selection procedure of each outcome is explained in Additional file 1. For both PA and SB outcomes, the Gamma model with the log link function was selected. In total, five separate models (i.e., total PA, PA intensities, PA domains, total SB, SB domains) were analysed. In order to assess the effect of the lockdown on total PA and SB, a model with total PA and one with SB as outcome variable and time as predictor variable was fitted. To inspect the lockdown effect in the different domains (PA and SB) or intensities (PA), the same model was fitted but with the domains or intensities as a categorical predictor variable together with an interaction term between time and domains or intensities. Significance of main and interaction effects of the categorical variables consisting of more than two categories were checked using Wald *Chi²* tests (Anova function from the R package car [29]). Contrasts were constructed (test Interactions function from the R package phia [30]) to inspect statistical differences between T0 and T-2, T-1, T1, T2 of each domain and intensity, respectively. Data visualisation was performed using the R packages ggplot2 [31] and sjPlot [32], based on the predicted values of the response variable. More detailed information on the statistical analysis procedure can be found in Additional file 2.

## Results

Of the initial 2,220 secondary school teachers that were included at the start of the larger study (T-2; Sep/Oct, 2019), 1,741 consented to be recontacted and provided their e-mail address. Of these, 830 filled in the questionnaire at T0 (Jan/Feb, 2020), which corresponds to a response rate of 47·7%. In total, 624 participants were included at T0, as 206 participants were excluded from the sample in line with the exclusion criteria as described above (i.e., due to injuries and/or following a diet (*n* = 172), sick leave (*n* = 8), not working in secondary education (*n* = 1), outliers for total PA (*n* = 8), outliers for SB (*n* = 9) and missing values for PA (*n* = 17). During lockdown-exposure (T1; Mar/Apr, 2020), the same 1,741 participants were invited to fill in the questionnaire again. Of these, 646 participants filled in the questionnaire (i.e., 37·1% response rate), of which 499 remained after exclusion (i.e., due to injuries and/or following a diet (*n* = 84), sick leave (*n* = 3), not working in secondary education (*n* = 10), outliers for total PA (*n* = 10), outliers for total SB (*n* = 41) and missing values (*n* = 13).

### Sample characteristics

The final baseline (T0) sample consisted of 624 participants (77·2% females) with a mean age of 43·3 ± 10·3 years and a mean BMI of 25·2 ± 4·6 kg/m² at baseline (T0). More detailed information regarding sample characteristics can be found in Table 1.

**Table 1:** Sample characteristics at baseline (T0; %, mean ± SD)

	*n* = 624
Sex (% females)	77·2
Age (years)	43·3 ± 10·3
Diploma (%)	
Secondary school degree	0·3
Post-secondary school degree	2·1
Bachelor’s degree	53·5
Master’s degree	42·9
PhD degree	1·1
Having an extra job (%)	11·1
Marital status (%)	
Single	12·9
Married	53·4
Unmarried	3·9
Living together with partner	22·5
Divorced	6·8
Widowed	0·5
Having children (%)	74·3
Ethnicity (%)	
White, European	99·0
White, other	0·5
North-African	0·5
Education network (%)	
Flemish community schools	47·7
Subsidised free schools	47·9
Subsidised public schools	3·7
Mixed	0·6
Total working hours (hours/week)	38·3 ± 18·2
BMI (kg/m²)	25·2 ± 4·6
Smoking status (% non-smoker)	90·4

*SD* standard deviation, *BMI*  body mass index

### Representativeness of the sample at baseline (T0)

To determine representativeness, our sample was compared to the general secondary teacher population (see Table C1, Additional file 3) [33]. . Significant differences were found for sex (sample vs. population: 22·8% vs. 35·1% males; 77·2% vs. 64·9% females, *p* < 0·001) and education network (sample vs. population: Flemish community schools: 47·7% vs. 22·5%; subsidised free schools: 47·9% vs. 68·0%; subsidised public schools: 3·7% vs. 9·4%; *p* < 0·001). Regarding age, no significant differences were found for the age categories 30–39 years (*p* = 0·595), 40–49 years (*p* = 0·554), 50–59 years (*p* = 0·268) and plus 60 years (*p* = 0·342), whereas a significant difference was found for secondary school employees between 20 and 29 years (sample vs. population: 10·0% vs. 14·8%; *p* = 0·0008).

### Drop-out analysis between T0 and T1

The drop-out rate between T0 and T1 was 20·0%. Significant differences at baseline (T0) were found between the retention and drop-out group regarding age (44·5 ± 10·4 vs. 41·8 ± 9·9; *p* = 0·002) and teaching hours per week (19·9 ± 4·5 vs. 20·6 ± 4·2; *p* = 0·035). No differences at baseline (T0) between the retention and drop-out group were found for sex (*p* = 0·930), diploma (*p* = 0·271), having an extra job (*p* = 0·993), marital status (*p* = 0·587), having children (*p* = 0·448), ethnicity (*p* = 0·228), education network (*p* = 0·615), BMI (*p* = 0·306), smoking status (*p* = 0·092), total PA (*p* = 0·727) and total SB (*p* = 0·419).

The second analysis showed only one significant difference at T1 between the participants of whom we had data at T0 and T1, and participants of whom we only had data at T1 regarding marital status (single = 12·2%, unmarried = 3·7%, married = 53·7%; living together with partner = 22·4%; divorced = 8·0%, widowed = 0·0% vs. single = 5·0%, unmarried = 2·2%, married = 54·7%; living together with partner = 29·5%; divorced = 6·5%, widowed = 2·2% vs. *p* = 0·008). No differences at T1 between both groups were found for age (*p* = 0·637), sex (*p* = 0·534), diploma (*p* = 0·584), having an extra job (*p* = 0·455), having children (*p* = 0·530), ethnicity (*p* = 0·693), education network (*p* = 0·627), BMI (*p* = 0·118), smoking status (*p* = 0·860), total PA (*p* = 0·538) and total SB (*p* = 0·198).

### Changes in PA and SB during lockdown

All models included random intercepts for the participants. The estimates of both fixed and random effects of each model can be found in Tables A1-A5 in Additional file 1. Possible clustering effects of school were checked by adding the school level to the models. As results showed that there was hardly any variance explained by this level, the cluster of school was not included. Hence, only two levels (repeated measures clustered within participants) were included in the final models. Based on the contrasts, Table 2 and the text below report on the adjusted means and standard errors of link for each of the outcomes pre- and during lockdown (crude means and standard deviations for all measurements can be found in Additional file 4, Table D1).

**Table 2:** Multilevel gamma models with log link function results for physical activity and sedentary behaviour pre-lockdown and during lockdown (Adjusted mean ± SE)

	Pre-lockdown (T0)		During lockdown (T1)		
	Adjusted mean	SE of link	Adjusted mean	SE of link	*p*-value
Physical activity (min/week)					
Total	1077·97	0·03	1185·63	0·03	0·047
**Domain-specific PA**					
Work-related	417·09	0·05	128·28	0·05	< 0·0001
Transport-related	189·25	0·05	151·18	0·05	0.005
Domestic and garden	393·14	0·05	701·29	0·05	< 0·0001
Leisure-time	176·27	0·05	307·61	0·05	< 0·0001
**Intensity-specific PA**					
Walking	430·63	0·05	399·18	0·05	1·0
Moderate-intensity	760·41	0·05	977·47	0·05	0·0010
Vigorous-intensity	53·43	0·05	46·96	0·06	0·57
**Sedentary behaviour** **(min/week)**					
Total	2992·36	0·01	3964·50	0·02	< 0·0001
**Domain-specific SB**					
Work-related	671·22	0·04	1333·18	0·04	< 0·0001
Transport-related	397·96	0·04	107·57	0·05	< 0·0001
Leisure-time	2065·50	0·04	2657·22	0·04	0.0004

Multilevel gamma models with log link functions were conducted for all outcome variables

*SE *Standard error, *PA* Physical activity, *SB* Sedentary behaviour

### Changes in physical activity

#### Total physical activity

**Fig. 2:**
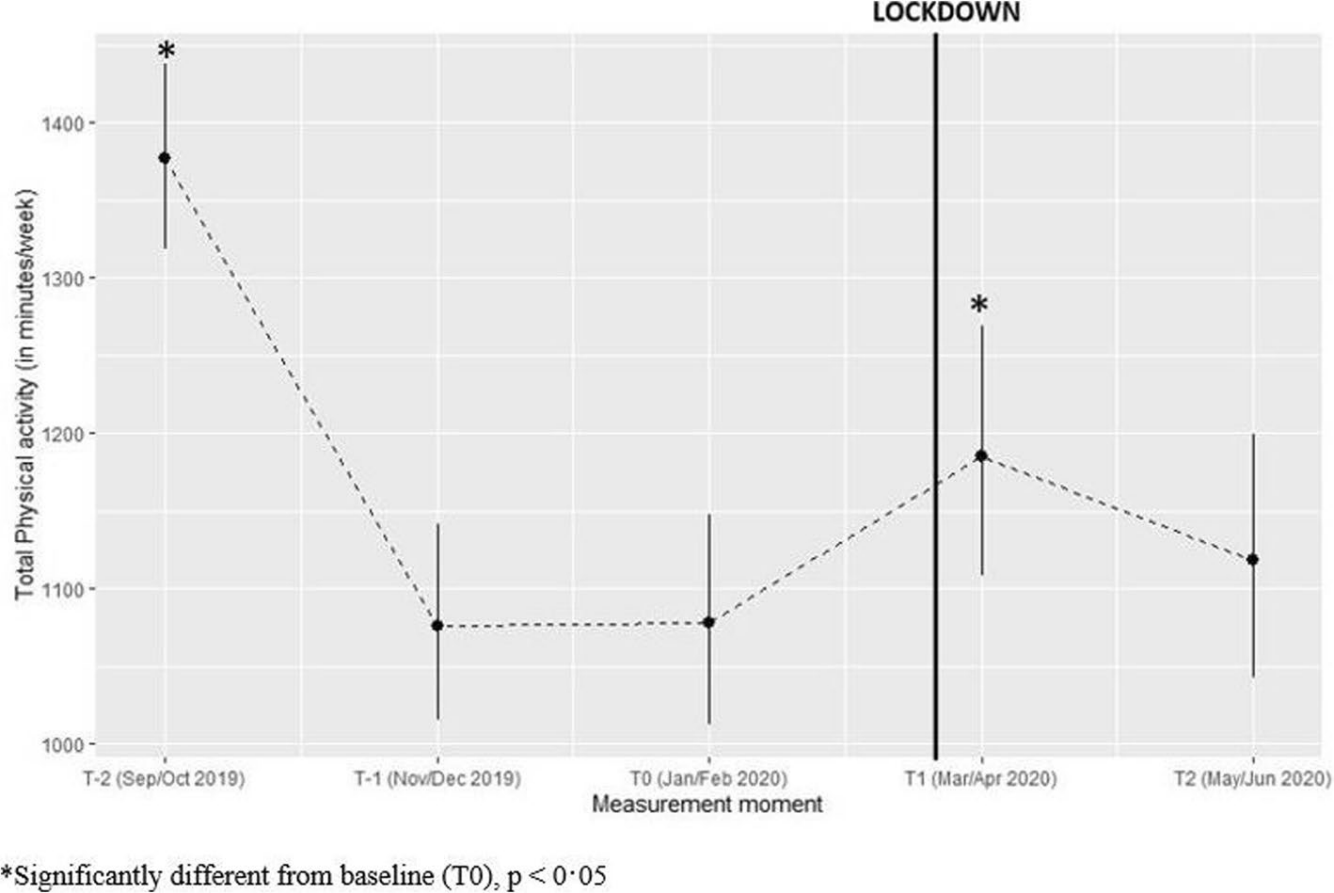
Predicted values for total physical activity (minutes/week) with confidence intervals for each measurement (Values estimated using gamma model with log link function)

On average, participants were 108 min/week more active during lockdown as compared to pre-lockdown (*p* = 0·047) (see Fig. 2).

#### Domain-specific physical activity

**Fig. 3:**
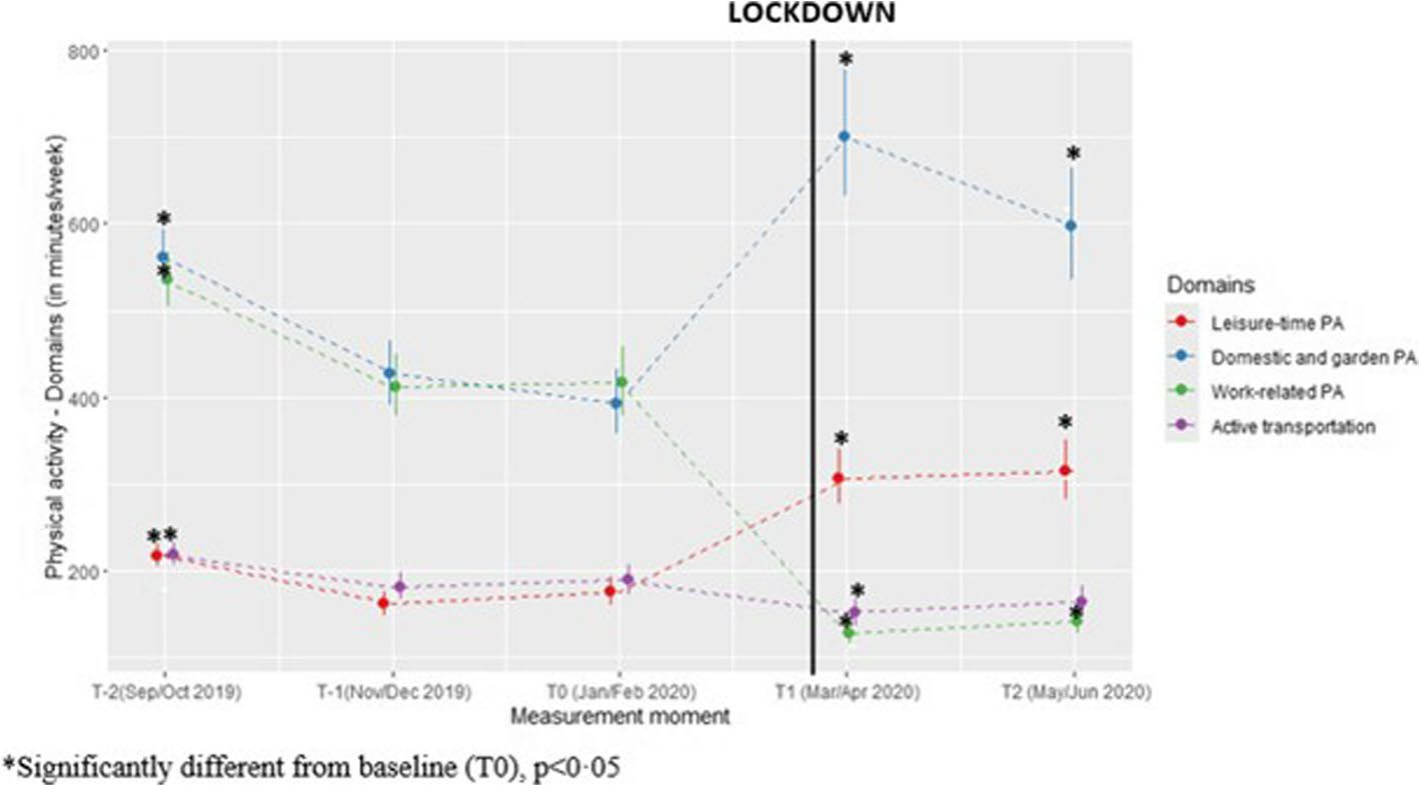
Predicted values for domain-specific physical activity (minutes/week) with confidence intervals for each measurement. (Values estimated using gamma model with log link function)

On average, participants were 308 min/week more occupied in domestic and garden PA and 131 min/week more occupied in leisure-time PA (both *p* < 0·0001) during lockdown compared to pre-lockdown. In contrast, participants showed 289 min/week less work-related physical activity (*p* < 0·0001) and 38 min/week less transport-related physical activity (*p* = 0·005) during lockdown compared to pre-lockdown (see Fig. 3).

#### Intensity-specific physical activity

**Fig. 4:**
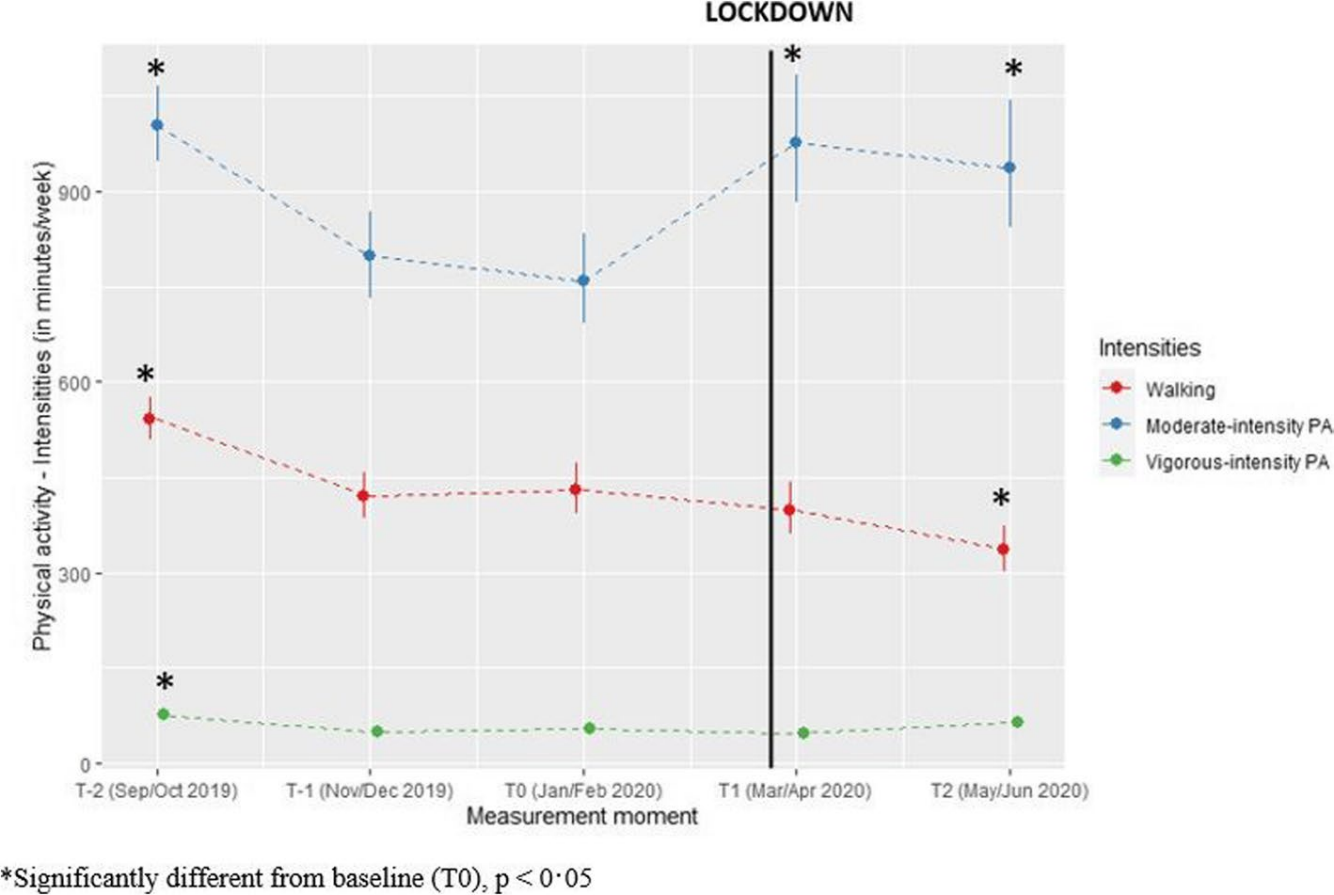
Predicted values for intensity-specific physical activity (minutes/week) with confidence intervals for each measurement. (Values estimated using gamma model with log link function)

On average, participants were 217 min/week more physically active at moderate intensity (*p* = 0·0010) during lockdown compared to pre-lockdown. No significant differences regarding walking (*p* = 1·0) and vigorous intensity PA (*p* = 0·57) between during lockdown and pre-lockdown were found (see Fig. 4).

### Changes in sedentary behaviour

#### Total sedentary behaviour

**Fig. 5:**
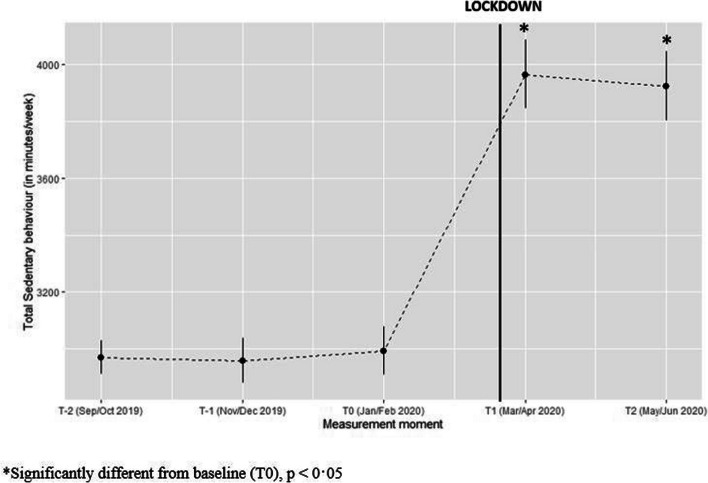
Predicted values for total sedentary behaviour (minutes/week) with confidence intervals for each measurement. (Values estimated using gamma model with log link function)

On average, participants were 972 min/week more sedentary (*p* < 0·0001) during lockdown as compared to pre-lockdown (see Fig. 5).

#### Domain-specific sedentary behaviour

**Fig. 6:**
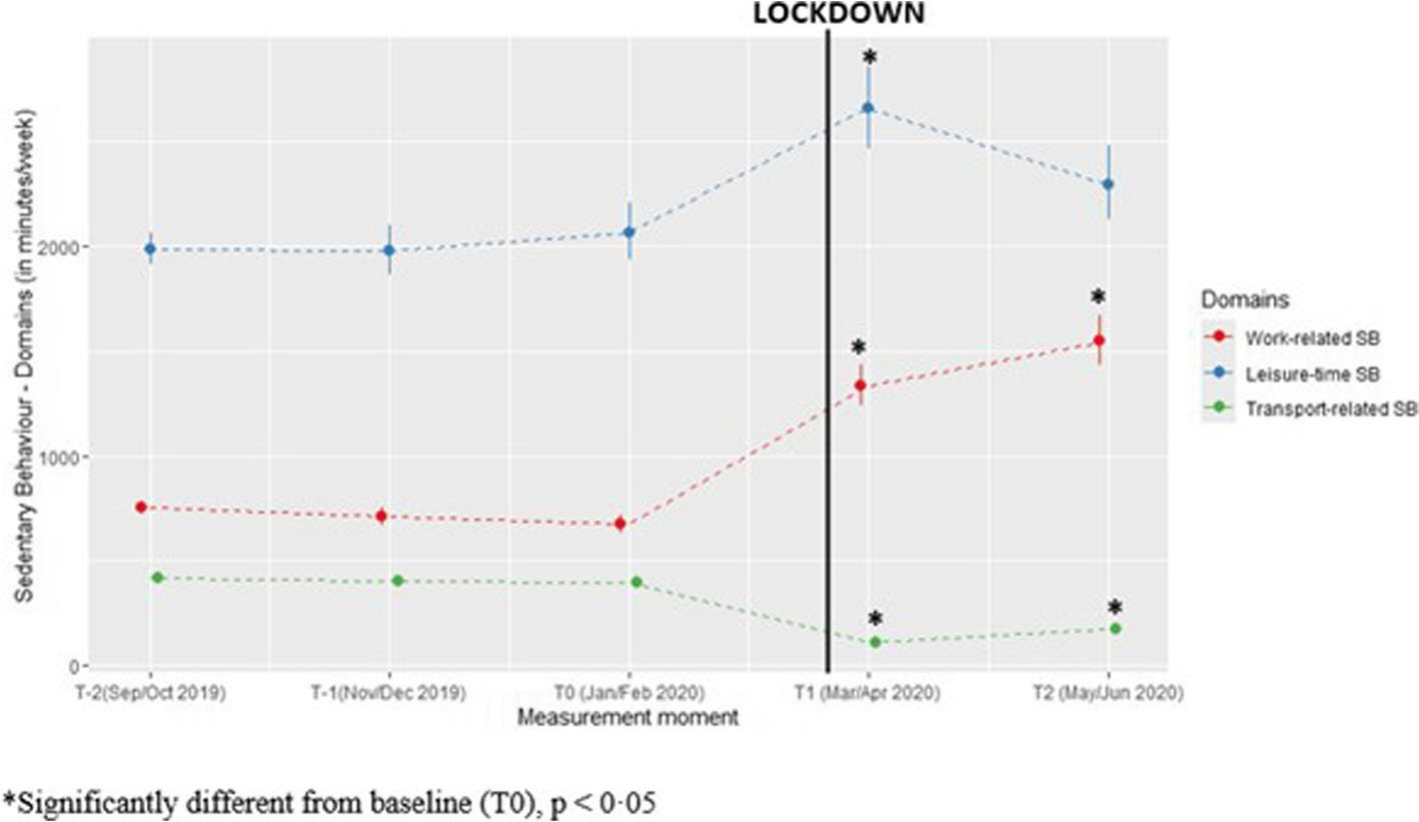
Predicted values for domain-specific sedentary behaviour (minutes/week) with confidence intervals for each measurement. (Values estimated using gamma model with log link function)

On average, participants were 662 min/week more sedentary during their work (*p* < 0·0001) and 592 min/week more sedentary during their leisure-time (*p* = 0·0004) during lockdown compared to pre-lockdown. In contrast, participants were 290 min/week less sedentary during transport (*p* < 0·0001) during lockdown compared to pre-lockdown (see Fig. 6).

## Discussion

### Summary of the findings

The most important finding is that, during the first Belgian COVID-19 lockdown (installed on March 18, 2020) both total PA (+ 108 min/week, i.e., 1·8 h/week) and total SB (+ 972 min/week, i.e., 16·2 h/week) had increased in Flemish secondary school teachers. The observed increase in total PA was due to increases in both domestic and garden PA, and leisure-time PA (when considering PA domain) and in moderate intensity PA (when considering PA intensity). In contrast, walking and vigorous PA remained stable over time, while work-related PA and transport-related PA decreased. The observed increase in total SB was due to increases in both work-related SB and leisure-time SB, while transport-related SB decreased.

### Strengths and limitations

An important strength of the present study is that we were able to use a prospective cohort design with repeated measures to evaluate the effect of the lockdown on PA and SB in secondary school teachers. Compared to other, mostly retrospective cross-sectional research, these prospective cohort data are unique and important to estimate the impact of COVID-19-induced lockdown measures on energy expenditure behaviour. The repeated pre- and during-lockdown measurements also allowed us to control (partially) for confounding, as for obvious reasons, it was not possible to compare against a control group. Second, this study provides more detailed information on the domain (PA and SB) and intensity (PA) level. The added value of this approach can be illustrated by the statistical outcomes of this study. Despite the fact that the models regarding the impact of the lockdown on total PA and total SB each explained a large amount of variance (total PA: 46·8%; total SB: 51·2%), our findings show that only a very small amount of variance was explained by the fixed effects (i.e., time) (total PA: 2%; total SB: 8·6%). When conducting the analyses per domain (PA and SB) and intensity (PA) level, the amount of variance explained by the fixed effects (i.e., time) increased considerably (i.e., domain-specific PA: 13·6%; intensity-specific PA: 37·9%; domain-specific SB: 49·9%). This underscores the importance of investigating domain- and/or intensity-specific PA and SB. Third, a relatively low drop-out rate of 20·0% between T0 and T1 was observed, while rather small significant baseline differences between the retention and drop-out group were found for age and teaching hours per week. This suggests limited drop-out bias during the course of the study. However, it should be mentioned that, of the 624 participants included at T0, only 353 (i.e., 56.6%) were included at T1. Furthermore, a selection bias may have been present upon recruitment as our sample was not representative for sex, the youngest age category and education network. Unfortunately, no other population-level data were available to check representativeness. A second limitation is the fact that we only included secondary school teachers. As mentioned above, this study is part of a larger longitudinal study among Flemish secondary school teachers. This means that, although societally relevant, our findings may not be generalized to other populations or occupations. For example, we expect that the increase in SB would be less dramatic in people performing a white-collar (desk, managerial or administrative) vs. blue-collar (labour) job. Third, although the IPAQ protocol includes truncation rules (i.e., topping off extreme or unrealistic values) [24], we decided to not apply these truncations, as this would have resulted in statistical issues concerning model-fit. However, we do not expect this to have influenced the results as this way of processing was applied systematically across all time points. Furthermore, it should be mentioned that total light-intensity PA, in which walking is just one component, is not questioned in the IPAQ. Therefore, the results on total PA in this study only represents walking and moderate-to-vigorous-intensity PA. Fourth, recall and social desirability bias are likely to be present as PA and SB were subjectively measured by means of self-report questionnaires [34]. As the same measurement tools were used across all time points, we expect this to have resulted in a systematic rather than unsystematic error, limiting biases on the established effects over time. Although systematic across time points, the omitted truncations and the used self-reports probably did cause overestimations of PA in absolute figures. Our findings indeed show that, on average, secondary school teachers engaged in 877·4 min/week (i.e., 2·1 h/day; see Additional file 4, Table D1) of moderate-to-vigorous-intensity PA (MVPA) at baseline, which is almost six times the minimal amount of 150 min/week recommended by the World Health Organization (WHO) [35]. Given that 35·9% of the Flemish adult population is not meeting the guidelines [36], the reported PA levels seem quite unrealistic and overestimated. This typical overestimation of PA due to the use of self-reports is well-acknowledged and described in the literature [24, 37]. Hence, the absolute values of PA (and possibly also SB) in the present study should be interpreted with great caution.

### Interpretation and significance of the findings

Our findings are in line with those of Constandt and colleagues [19], reporting a general increase in both PA and SB in the general Belgian adult population. As mentioned, due to methodological limitations of the latter study, no comparison could be made concerning the duration and intensity of PA, nor in which domains PA was performed. In contrast, the review by Stockwell and colleagues [18] showed that, across several countries and several populations, PA levels decreased during the lockdown. This discrepancy may be explained by differences in study populations as well as by country-specific differences in lockdown measures installed by the different governments. For example, the confinement measures in Italy or Spain, where people were not allowed to leave their homes except for necessities while outdoor PA was prohibited, were more strict than those in Belgium, where people were still allowed and even stimulated to move around and engage in outdoor yet restricted and non-organised PA (e.g., walking, cycling only within the “family-bubble”) [18]. Although we may applaud that people were stimulated to engage in healthy behaviour, such as PA, no attention was raised to discourage unhealthy behaviour, such as SB. Indeed, our results even show that the increase in SB was 9 times as high as the increase in PA. The disproportionate increase of SB may have detrimental (acute) effects on health outcomes, especially when they sustain in the long run [2]. With their meta-analysis, including over one million people, Ekelund and colleagues [38, 39] demonstrated a clear dose-response relationship between increased sitting time and all-cause mortality. In case of lower PA levels, the risk of sitting-induced mortality may be as high as that of smoking and obesity [39]. Interestingly, the same study revealed that engaging in MVPA for at least one hour/day may attenuate or even eliminate the detrimental effects of sitting for more than eight hours/day [38, 39]. So, PA becomes even more relevant as long periods of sitting time during lockdown (e.g., due to homework) may be unavoidable.

While decreases were observed in other studies [18], walking was not impacted by the lockdown in our sample of secondary school teachers. This peculiar finding can be explained by the fact that teachers typically stand or walk around while performing their job. More detailed analysis (data not shown) indeed revealed that walking during leisure-time increased, while work-related walking decreased, cancelling each other out. This finding seems fairly logic as schools were closed and teachers were asked to work from home. On the domain level, this explains both the decrease in work-related PA and the overproportionate increase in work-related SB. Obviously, this same mechanism explains the reduced transport-related PA and SB.

Another interesting finding is that, unlike a significant increase in moderate-intensity PA, vigorous-intensity PA remained stable during the lockdown. As previous research highlighted the importance of sport infrastructure for sport participation rates [40], one might expect vigorous PA to have decreased due to the cancellation of organised sports and group-based physical activities (typically including higher-intensity activities and competition sports) as sport clubs were closed. This may indicate that the participants, as stimulated by the authorities, replaced these latter activities with other (self-organised) vigorous-intensity PA, such as running or cycling. This ‘compensatory effect’ was reported in previous research, suggesting that an increase in PA in one domain may result in compensatory changes in another one [41]. A systematic review by Swelam, Verswijveren [42] on the other hand, concluded that evidence regarding these compensatory effects is mixed.

Finally, as also discussed in previous research [21, 43], the effects of the lockdown on PA and SB may be respectively over- and underestimated due to the relatively warm and sunny weather during the first lockdown period. Baseline (T0) measurements took place from Jan 27 to Feb 11, 2020 (i.e., 16-day measurement period), with maximum daily temperatures up to 13 °C, only 2 days with at least 5 h of sun per day, and 11 precipitation days [44]. In contrast, T1 measurements took place from Mar 23 to Apr 7, 2020 (i.e., 16-day measurement period), with maximum daily temperatures up to 23 °C, 9 days with at least 10 h of sun per day, and zero precipitation days [44]. This sunny, dry, and relatively warm weather during the first lockdown may have biased our findings by causing our participants to be more physically active. The extent of bias becomes clearer when interpreting the domain-specific PA models. As explained above, due to the greater amount of explained variance by the fixed time effect, it becomes more relevant to interpret the intensity- and domain-specific PA models compared to the total PA model. For example, when interpreting the total PA model, we observed a relatively larger decrease in total PA from September to November 2019, and a relatively smaller increase of total PA from January to March 2020 (i.e., during the lockdown). So, one might argue that the observed increase in PA may not be entirely attributed to the lockdown measures per se and that it is just a natural (including seasonal) fluctuation. However, when splitting up our models, we observed different domain-specific PA effects going in the opposite directions, diminishing the overall effect for total PA. Indeed, in contrast to the total PA model, the domain-specific models showed relatively larger increases or decreases (depending on the domain) in PA during the lockdown compared to other natural (including seasonal) fluctuations (indicated by the control measurements). This suggests that seasonal and/or weather effects were rather limited and that a great part of the effect can be attributed to the lockdown measures. It is also noteworthy that leisure-time SB increased substantially, despite the good weather circumstances. This is probably due to a lack of other (social) activity options, such as going out with friends, or participating in cultural events.

### Recommendations for policy and research

On the policy level, one should be aware that installing such lockdown measures come with a price. In the present study, participants’ SB increased by 16·2 h/week (i.e., 2·3 h/day). More specifically, secondary school teachers went from 7·1 to 9·4 h/day of being sedentary. As highlighted above, this may have detrimental short- and long-term effects on people’s physical and mental health, especially in people being less than one hour/day physically active [38, 39]. Taking a probable and considerable overestimation of PA into account, it is highly doubtable that many participants met this one hour/day threshold. It is clear that adequate levels of PA and reduced levels of SB are crucial components of a healthy lifestyle and are related to various comorbidities and mortality [38, 39]. As a government, it is crucial to sensitize, promote, and facilitate sufficient MVPA and/or walking (as measured in the present study), but likewise to discourage SB (e.g., teachers may be advised to teach their online classes while standing or walking), even in such difficult times of pandemic-induced lockdown measures.

## Conclusion

The COVID-19 lockdown was associated with an increase in PA of 108 min/week (i.e., 1·8 h/week) and an increase in SB of 972 min/week (i.e., 16·2 h/week) in secondary school teachers. Although the Belgian authorities encouraged people to engage in PA, the discouragement of SB was lacking. Promoting a physically active as well as a non-sedentary lifestyle is highly important during a pandemic and should be considered a priority for governments installing such lockdown measures.

### Supplementary Information

Additional file 1: Appendix A. Model selection procedure.

Additional file 2: Appendix B. Statistical analysis plan.

Additional file 3: Appendix C. Sample representativeness.

Additional file 4: Appendix D. Crude means and standard deviations.

## Data Availability

Data are available upon reasonable request (yanni.verhavert@vub.be).

## Abbreviations

PA: physical activity
SB: sedentary behaviour
BMI: body mass index
IPAQ: International Physical Activity questionnaire
BIC: Bayesian Information Criterion
MVPA: moderate-to-vigorous physical activity
WHO: World Health Organization
