# Supplementary material for: The impact of COVID-19 lockdown on physical activity and sedentary behaviour in secondary school teachers: a prospective cohort study
Source: BMC Public Health. 2024 Jun 5;24:1508. doi: 10.1186/s12889-024-18954-4 (PMC11155126; doi:10.1186/s12889-024-18954-4)
Supplement: Supplementary file 1 — Additional file 1: Appendix A. Model selection procedure. [file 12889_2024_18954_MOESM1_ESM.pdf]

APPENDIX A: MODEL SELECTION PROCEDURE

MODEL SELECTION - TOTAL PHYSICAL ACTIVITY

A gamma GLiM was constructed with total PA as outcome and time as a predictor variable. The gamma model with the log link function (BIC = 68675·5) was preferred over the Gaussian model with the log function (BIC = 96666·4), the gamma model with the inverse link function and the Gaussian model with the identity function. The latter two models did not fit the data due to convergence issues and the gamma model with the log link function yielded a significantly better model fit (*Chi² difference* = 27991, $p < 0{\cdot}0001$).

The selected model (fixed and random effects) was able to explain 47.6% of the total variance (i.e. conditional R^2). In particular, 2·1% of the variance was explained by the fixed effects (i.e. time; marginal R^2) and 45·5% by the random effects (i.e. participants; conditional intraclass coefficient). The latter signifies the importance of taking into account clustering in the data.

Table A1. MLM results for Total Physical activity using a gamma distribution and a log link function

Total Physical Activity

**Predictors**	**Estimates**	**p-value**
**Fixed Effects**		
Intercept	6·98	**<0.0001**
Time(T-2)	0·24	**<0.0001**
Time (T-1)	-0·001	0.97
Time (T+1)	0·1	**0.010**
Time (T+2)	0·04	0.34
**Random Effects**		
σ^2_ϵ	0·31	
σ^2_{u0}	0·27	
ICC	0·46	
N_{id}	2015	
Observations	4211	
Marginal R^2 / Conditional R^2	0·021 / 0·476	

Abbreviations: σ^2_ϵ *= residual variance,* σ^2_{u0} *= variance component for between participant variability, ICC = intraclass correlation coefficient*
Reference category = T0

MODEL SELECTION - DOMAIN-SPECIFIC PHYSICAL ACTIVITY

A gamma GliM was constructed with domain-specific PA as outcome and time and domain type of PA as a predictor variable. The model with the log link function (BIC = 84562·2) was preferred over the Gaussian model with the log function (BIC = 280301·1), the Gaussian model with the identity function and the gamma model with the inverse link function. The latter two models did not fit the data due to convergence issues and the gamma model with the log link function yielded a significantly better model fit (*Chi² difference* = 195739, $p < 0{\cdot}0001$).

The selected model (fixed and random effects) was able to explain 38.1% of the total variance (i.e., conditional R^2). In particular, 13·6% of the variance was explained by the fixed effects (i.e., time; marginal R^2) and 28·0% by the random effects (i.e., participants; conditional intraclass coefficient). The latter signifies the importance of taking into account clustering in the data.

Table A2. MLM results for Physical activity - Domains using a gamma distribution and a log link function

Physical Activity - Domains

**Predictors**	**Estimates**	**p-value**
**Fixed Effects**		
Intercept	5·17	**<0·0001**
Time(T-2)	0·21	**<0·0001**
Time (T-1)	-0·08	0·15
Time (T+1)	0·56	**<0·0001**
Time (T+2)	0·58	**<0·0001**
PA(Domestic and garden)	0·80	**<0·0001**
PA(Work-related)	0·86	**<0·0001**
PA(Transport-related)	0·07	0·229
Time(T-2):PA(Domestic and garden)	0·14	**0·036**
Time (T-1):PA(Domestic and garden)	0·16	**0·038**
Time(T+1):PA(Domestic and garden)	0·02	0·80
Time (T+2):PA(Domestic and garden)	-0·16	0·07
Time(T-2):PA(Work-related)	0·03	0·60
Time(T-1):PA(Work-related)	0·07	0·37
Time(T+1):PA(Work-related)	-1·74	**<0·0001**
Time(T+2):PA(Work-related)	-1·66	**<0·0001**
Time(T-2):PA(Transport-related)	-0·06	0·34
Time(T-1):PA(Transport-related)	0·04	0·58
Time(T+1):PA(Transport-related)	-0·78	**<0·0001**
Time(T+2):PA(Transport-related)	-0·72	**<0·0001**
**Random Effects**		
σ_{ϵ}^{2}	1·18	
σ_{u0}^{2}	0·47	
ICC	0·28	
N_{id}	2015	
Observations	16844	
Marginal R^2 / Conditional R^2	0·136 / 0·381	

Abbreviations: σ_{ϵ}^{2} = residual variance, $\sigma_{u0}^{2} =$ variance component for between participant variability ICC = intraclass correlation coefficient
Reference categories = T0 and Leisure-time PA

MODEL SELECTION - INTENSITY-SPECIFIC PHYSICAL ACTIVITY

A gamma GLiM was constructed with intensity-specific PA as outcome and time and intensity type of PA as a predictor variable. The model with the log link function (BIC = 77024·0) was preferred over the Gaussian model with the identity function (BIC = 197367·9), the Gaussian model with the log function and the gamma model with the inverse link function. The latter two models did not fit the data due to convergence issues and the gamma model with the log link function yielded a significantly better model fit (*Chi² difference* = 120344, $p < 0·0001$). The selected model (fixed and random effects) was able to explain 63·1% of the total variance (i.e., conditional R^2). In particular, 37·9% of the variance was explained by the fixed effects (i.e., time; marginal R^2) and 25·1% by the random effects (i.e. participants; conditional intraclass coefficient). The latter signifies the importance of taking into account clustering in the data.

Table A3. MLM results for Physical activity - Intensities using a gamma distribution and a log link function

Physical Activity - Intensities

Predictors	Estimates	p-value
**Fixed Effects**		
Intercept	6·06	**<0.0001**
Time(T-2)	0·23	**<0.0001**
Time (T-1)	-0·03	0.66
Time (T+1)	-0·08	0.24
Time (T+2)	-0·25	**0.00020**
PA(Moderate-intensity)	0·57	**<0.0001**
PA(Vigorous-intensity)	-2·09	**<0.0001**
Time(T-2):Moderate-intensity PA	0·05	0.48
Time(T-1):Moderate-intensity PA	0·07	0.35
Time(T+1):Moderate-intensity PA	0·33	**0.00020**
Time(T+2):Moderate-intensity PA	0·46	**<0.0001**
Time(T-2):Vigorous-intensity PA	0·13	0.070
Time(T-1):Vigorous-intensity PA	-0·06	0.48
Time(T+1):Vigorous-intensity PA	-0·05	0.56
Time(T+2):Vigorous-intensity PA	0·42	**<0.0001**
**Random Effects**		
σ_ϵ^2	1·30	
σ_{u0}^2	0·88	
ICC	0·40	
N id	2015	
Observations	12633	
Marginal R^2 / Conditional R^2	0·379 / 0·631	

Abbreviations: σ_ϵ^2 *= residual variance,* $\sigma_{u0}^2 =$ *variance component for between participant variability ICC = intraclass correlation coefficient*
Reference categories = T0 and Walking

MODEL SELECTION - TOTAL SEDENTARY BEHAVIOUR

A gamma GliM was constructed with total SB as outcome and time as a predictor variable. The model with the log link function (BIC = 69671·0) was preferred over the Gaussian model with the log function (BIC = 97150·0), the Gaussian model with the identity function and the gamma model with the inverse link function. The latter two models did not fit the data due to convergence issues and the gamma model with log link function yielded a significantly better model fit (*Chi² difference* = 929·02, p < 0·0001).

The selected model (fixed and random effects) was able to explain 50·8% of the total variance (i.e., conditional R^2). In particular, 10·4% of the variance was explained by the fixed effects (i.e., time; marginal R^2) and 40·4% by the random effects (i.e., participants; conditional intraclass coefficient). The latter signifies the importance of taking into account clustering in the data.

Table A4. MLM results for Total Sedentary Behaviour using a gamma distribution and a log link function

Total Sedentary Behaviour

Predictors	Estimates	p-value
**Fixed Effects**		
Intercept	8·00	**<0·0001**
Time(T-2)	-0·01	0·55
Time (T-1)	-0·01	0·40
Time (T+1)	0·28	**<0·0001**
Time (T+2)	0·27	**<0·0001**
**Random Effects**		
σ_ϵ^2	0·07	
σ_{u0}^2	0·05	

ICC	0·45
N id	2015
Observations	4211
Marginal R^2 / Conditional R^2	0·104 / 0·508

Abbreviations: σ_ϵ^2 *= residual variance,* $\sigma_{u0}^2 =$ *variance component for between participant variability ICC = intraclass correlation coefficient*
Reference category = T0

MODEL SELECTION - DOMAIN-SPECIFIC SEDENTARY BEHAVIOUR

A gamma GLiM was constructed with domain-specific SB as outcome and time and domain type of SB as a predictor variable. The model with the log link function (BIC = 21489·81) was preferred over the Gaussian model with the log function (BIC = 3302547000·0), the Gaussian model with the identity function (BIC = 199874·0) and the gamma model with inverse link function. The latter model did not fit the data due to convergence issues and the gamma model with the log link function yielded a significantly better model fit (*Chi² difference* = 178084, $p < 0{\cdot}0001$).

The selected model (fixed and random effects) was able to explain 49·9% of the total variance (i.e., conditional R^2). In particular, 49·9% of the variance was explained by the fixed effects (i.e., time; marginal R^2) and 0·0% by the random effects (i.e., participants; conditional intraclass coefficient).

Table A5. MLM results for Sedentary Behaviour – Domains using a gamma distribution and a log link function

	Sedentary Behaviour - Domains	
**Predictors**	**Estimates**	**p-value**
**Fixed Effects**		
Intercept	6·51	**<0.0001**
Time(T-2)	0·12	**0.011**
Time (T-1)	0·06	**0.297**
Time (T+1)	0·67	**<0.0001**
Time (T+2)	0·83	**<0.0001**
SB(Leisure-time)	1·12	**<0.0001**
SB(Transport-related)	-0·52	**<0.0001**
Time(T-2):SB(Leisure-time)	-0·16	**0.016**
Time(T-1):SB(Leisure-time)	-0·10	0.195
Time(T+1):SB(Leisure-time)	-0·43	**<0.0001**
Time(T+2):SB(Leisure-time)	-0·73	**<0.0001**
Time(T-2):SB(Transport-related)	-0·07	0.315
Time(T-1):SB(Transport-related)	-0·05	0.478
Time(T+1):SB(Transport-related)	-1·99	**<0.0001**
Time(T+2):SB(Transport-related)	-1·67	**<0.0001**
**Random Effects**		
σ_ϵ^2	0·70	
σ_{u0}^2	0·00	
ICC	0·00	
N id	2015	
Observations	12633	
Marginal R^2 / Conditional R^2	0·499 / 0·499	

Abbreviations: σ_ϵ^2 *= residual variance,* $\sigma_{u0}^2 =$ *variance component for between participant variability ICC = intraclass correlation coefficient*
Reference categories = T0 and Work-related SB

Table A6. Goodness of fit per model - Log likelihood, Pseudo-R^2

	Log likelihood full	Log likelihood intercept	Pseudo-R^2
Total PA	-34308·5	-34365·8	0·002
Intensity-specific PA	-38431·7	-43355·9	0·114
Domain-specific PA	-42174·1	-44008·6	0·042
Total SB	-34806·3	-35167·2	0·010
Domain-specific SB	-10814·6	-14581·2	0·258

Abbreviations: PA = physical activity, SB = sedentary behaviour

Table A7: Changes in physical activity during lockdown - Chi²- and p-values

	Chi²-value	p-value
**Total PA**	114·58	**<0·0001**
**Domain-specific PA**		
Measurement moment	191·87	**<0·0001**
Domain	1379·40	**<0·0001**
Measurement moment* Domain	1174·36	**<0·0001**
**Intensity-specific PA**		
Measurement moment	89·359	**<0·0001**
Intensity	1964·385	**<0·0001**
Measurement moment* Intensity	77·883	**<0·0001**

Abbreviations: PA = physical activity

Table A8: Changes in sedentary behaviour during lockdown - Chi²- and p-values

	Chi²-value	p-value
**Total SB**	785·72	**<0·0001**
**Domain-specific SB**		
Measurement moment	349·7	**<0·0001**
Domain	2294·7	**<0·0001**
Measurement moment* Domain	1240·3	**<0·0001**

Abbreviations: SB = sedentary behaviour

Table A9. Physical activity & sedentary behaviour T-2, T-1, T0, T1 & T+1 – Adjusted mean ± SE

	T-2	T-1	T0	T1	T2
	Adjusted mean (SE of link)	Adjusted mean (SE of link)	Adjusted mean (SE of link)	Adjusted mean (SE of link)	Adjusted mean (SE of link)
**Physical activity (min/week)**					
Total PA	1376·79 (0·02)	1076·46 (0·03)	1077·97 (0·03)	1185·63 (0·03)	1118·12 (0·04)
**Domain-specific PA**					
Work-related PA	535·24 (0·03)	412·15 (0·04)	417·09 (0·05)	128·28 (0·05)	141·97 (0·05)
Transport-related PA	219·63 (0·03)	182·08 (0·04)	189·25 (0·05)	151·18 (0·05)	164·93 (0·05)
Domestic and garden PA	561·14 (0·03)	426·59 (0·04)	393·14 (0·05)	701·29 (0·05)	596·84 (0·05)
Leisure-time PA	218·15 (0·03)	162·34 (0·04)	176·27 (0·05)	307·61 (0·05)	314·96 (0·05)

**Intensity-specific PA**					
Walking	542·24 (0·03)	419·77 (0·04)	430·63 (0·05)	399·18 (0·05)	335·74 (0·05)
Moderate-intensity PA	1005·05 (0·03)	797·81 (0·04)	760·41 (0·05)	977·47 (0·05)	938·54 (0·05)
Vigorous-intensity PA	76·40 (0·03)	49·15 (0·05)	53·43 (0·05)	46·96 (0·06)	63·08 (0·06)
**Sedentary behaviour (min/week)**					
Total SB	2969·73 (0·01)	2957·46 (0·01)	2992·36 (0·01)	3964·50 (0·02)	3922·31 (0·02)
**Domain-specific SB**					
Work-related SB	755·41 (0·02)	710·08 (0·04)	671·22 (0·04)	1333·18 (0·04)	1545·81 (0·05)
Transport-related SB	419·42 (0·02)	398·81 (0·04)	397·96 (0·04)	107·57 (0·05)	172·16 (0·05)
Leisure-time SB	1985·65 (0·02)	1979·27 (0·04)	2065·50 (0·04)	2657·22 (0·04)	2295·86 (0·05)

Table A10: Differences in total PA & total SB compared to baseline (T0) - Chi²- and p-values

	**Chi²-value**	**p-value**
**Total PA**		
September - January	66·72	**<0·0001**
November - January	0·002	0·97
January - March	6·74	**0·047**
January - May	0·92	0·93
**Total SB**		
September - January	0·36	1·0
November - January	0·70	1·0
January - March	334·11	**<0·0001**
January - May	293·32	**<0·0001**

Abbreviations: PA = physical activity, SB = sedentary behaviour

Table A11: Differences in PA intensities & domains compared to baseline (T0) - Chi²- and p-values

	**Chi²-value**	**p-value**
**Intensity-specific PA**		
**September - January**		
Walking PA	20·51	**0·00010**
Moderate-intensity PA	31·25	**<0·0001**
Vigorous-intensity PA	44·93	**<0·0001**
**November - January**		
Walking PA	0·20	1·0
Moderate-intensity PA	0·71	1·0
Vigorous-intensity PA	1·91	1·0
**January - March**		
Walking PA	1·40	1·0
Moderate-intensity PA	15·79	**0·0010**
Vigorous-intensity PA	3·62	0·57
**January - May**		
Walking PA	14·37	**0·0030**
Moderate-intensity PA	10·44	**0·019**
Vigorous-intensity PA	5·55	0·20
**Domain-specific PA**		
**September - January**		
Work-related PA	25·00	**<0·0001**
Transport-related PA	9·14	**0·031**
Domestic and garden PA	52·37	**<0·0001**
Leisure-time PA	18·28	**0·0003**

**November - January**		
Work-related PA	0·04	1·0
Transport-related PA	0·47	1·0
Domestic and garden PA	2·10	1·0
Leisure-time PA	2·09	1·0
**January - March**		
Work-related PA	320·36	**<0·0001**
Transport-related PA	12·64	**0·005**
Domestic and garden PA	86·10	**<0·0001**
Leisure-time PA	78·16	**<0·0001**
**January - May**		
Work-related PA	256·52	**<0·0001**
Transport-related PA	4·53	0·33
Domestic and garden PA	41·85	**<0·0001**
Leisure-time PA	80·70	**<0·0001**

Abbreviations: PA = physical activity, SB = sedentary behaviour

Table A12: Differences in SB domains compared to baseline (T0) - Chi²- and p-values

	Chi²-value	p-value
**Domain-specific SB**		
**September - January**		
Work-related SB	6·53	0.14
Transport-related SB	1·29	1·0
Leisure-time SB	0·73	1·0
**November - January**		
Work-related SB	1·09	1·0
Transport-related SB	0·002	1·0
Leisure-time SB	0·62	1·0
**January - March**		
Work-related SB	130·43	**<0·0001**
Transport-related SB	445·56	**<0·0001**
Leisure-time SB	17·59	**0·0004**
**January - May**		
Work-related SB	183·52	**<0·0001**
Transport-related SB	184·63	**<0·0001**
Leisure-time SB	2·95	0·77

Abbreviations: SB = sedentary behaviour
