# Supplementary material for: The impact of COVID-19 lockdown on physical activity and sedentary behaviour in secondary school teachers: a prospective cohort study
Source: BMC Public Health. 2024 Jun 5;24:1508. doi: 10.1186/s12889-024-18954-4 (PMC11155126; doi:10.1186/s12889-024-18954-4)
Supplement: Supplementary file 2 — Additional file 2: Appendix B. Statistical analysis plan. [file 12889_2024_18954_MOESM2_ESM.pdf]

APPENDIX B: STATISTICAL ANALYSIS PLAN

All data were analysed using R (R core Team, 2019; R Studio version 3.6.2) and SPSS (version 27). *P*-values < 0·05 were considered to be statistically significant, whereas *p*-values between 0·05 and 0·10 were considered marginally significant. Drop-out analyses between baseline (T0) and the primary endpoint (T1) were conducted to assess possible selection bias of the retention group. To this end, independent samples *t*-tests, Mann-Whitney U tests and *chi²* tests were conducted to detect possible differences between the drop-out group and retention group regarding total PA, total SB, sex, age, ethnicity, marital status, having children, smoking status, diploma, occupation, having an extra job, education network and BMI. Representativeness of the sample at baseline (T0) was assessed by conducting two proportions z-tests.

The PA scale scores were non-normally distributed with continuous, positively skewed non-negative values. Gamma and Gaussian generalized linear mixed models were applied using the R package lme4.[15] The corresponding *p*-values were extracted using the R package lmerTest.[29] To decide upon the link functions (i.e., log, inverse or identity), Bayesian Information Criterion (BIC) values were compared and a likelihood ratio test was performed (lrtest function of the R package lmtest[16]). In case of convergence problems or a singular fit, greater accuracy for evaluating the adaptive Gauss-Hermite approximation to the log-likelihood was allowed. For this, the nAGQ-argument of the glmer function was set to two or more, producing greater accuracy in the evaluation of the log-likelihood. Note that this argument defaults to 1, corresponding to the Laplace approximation. After adjusting the nAGQ-argument, models that still not converged, were not further taken into account.

The SB scales also contained non-negative continuous values, but with less severe skewness. Therefore, models with different variance and link functions were compared (i.e., Gaussian with identity, Gaussian with log, Gamma with log and Gamma with inverse) using both the BIC value and the likelihood ratio test. In line with the PA scale scores, after adjusting the nAGQ-argument, models that still not converged, were not further taken into account.

For all models, the deviance residuals were inspected to verify that the selected model was suitable to analyse the data. In addition, model quality for each model was assessed and details about these results can be found in appendix B. To assess whether or not a model with predictors is significantly better than a model without predictors, goodness of fit (pseudo-R²) of the final models was calculated as follows:

$$1 - \frac{\text{Log likelihood full}}{\text{Log likelihood intercept}}$$

To assess to what extent (in %) the variance of the generalized mixed effects models is explained by the random and fixed effects, goodness of fit measures (pseudo-R^2) of the final models were calculated by calculating the marginal and condition R^2-values. Note that the marginal R^2-value indicates how much of the variance is explained by the fixed effects, whereas the conditional R^2 shows how much of the variance is explained by both the fixed and random effects. Both values are computed using the r2_nakagawa function of the R package performance.[30] The proportion of variance explained by solely the random effects was calculated using the intraclass correlation (ICC function of the R package performance[30]) but could also be calculated by subtracting the previous two measures.

Preliminary analyses checked if a three level model was advised (repeated measures clustered within participants, participants clustered within schools) using graphical representations and by inspecting the amount of variance explained by each cluster. If necessary, one (or both) levels were dropped.

In total, five separate models were analysed. In order to assess the effect of the lockdown on total PA, a model with total PA as outcome variable and time as predictor variable was fitted. To inspect the lockdown effect in the different domains or intensities of PA, the same model was fitted but with the domains or intensities as a categorical predictor variable together with an interaction term between time and domains or intensities. Significance of main and interaction effects of the categorical variables consisting of more than two categories were checked using Wald Chi^2 tests (Anova function from the R package car[17]). Contrasts were constructed (test Interactions function from the R package phia[18]) to inspect the statistical difference between T0 and T1 of each domain and intensity, respectively.

Likewise, when studying the effect of the lockdown on total SB, a model with total SB as outcome and time as a predictor variable was fitted. To inspect the lockdown effect in the different domains of SB, both the domains and the interaction term between domains and time were included as predictor variables. Contrasts were again used to inspect the statistical difference between T0 and T1 for each domain, separately.

Data visualisation was performed using the plot model function of R packages ggplot2[19] and sjPlot[20], based on the predicted values of the response variable. Statistical procedures and analyses have been guided and checked by a statistician.
