# Supplementary material for: The impact of COVID-19 lockdown on physical activity and sedentary behaviour in secondary school teachers: a prospective cohort study
Source: BMC Public Health. 2024 Jun 5;24:1508. doi: 10.1186/s12889-024-18954-4 (PMC11155126; doi:10.1186/s12889-024-18954-4)
Supplement: Supplementary file 3 — Additional file 3: Appendix C. Sample representativeness. [file 12889_2024_18954_MOESM3_ESM.pdf]

APPENDIX C: SAMPLE REPRESENTATIVENESS

Table C1. Representativeness of the sample at baseline (T0)

			**X²**	**p-value**
	**Sample (n = 614)**[$]	**Population (n = 77,802)**		
**Sex (n (%))**				
Males	140 (22·8%)	27,300 (35·1%)	40·44	<0·0001
Females	474 (77·2%)	50,502 (64·9%)	40·44	<0·0001
	**Sample (n = 613)**[$]	**Population (n = 77,802)**		
**Age (years) (n (%))**				
20-29	61 (10·0%)	11,508 (14·8%)	11·33	0·00080
30-39	186 (30·3%)	22,844 (29·4%)	0·28	0·60
40-49	171 (27·9%)	20,876 (26·8%)	0·35	0·55
50-59	154 (25·1%)	18,071 (23·2%)	1·22	0·27
+60	41 (6·7%)	4,503 (5·8%)	0·90	0·34
	**Sample (n = 504)**[$]	**Population (n = 77,802)**		
**Education network (n (%))**				
Flemish community schools	242 (47·7%)	17,471 (22·5%)	186·91	<0·0001
Subsidised free schools				
Subsidised public schools	243 (47·9%)	52,937 (68·0%)	90·33	<0·0001
	19 (3·7%)	7,349 (9·4%)	18·93	<0·0001

[$]Sample sizes may differ from the initial 624 due to missing data
