# Supplementary material for: The impact of COVID-19 lockdown on physical activity and sedentary behaviour in secondary school teachers: a prospective cohort study
Source: BMC Public Health. 2024 Jun 5;24:1508. doi: 10.1186/s12889-024-18954-4 (PMC11155126; doi:10.1186/s12889-024-18954-4)
Supplement: Supplementary file 4 — Additional file 4: Appendix D. Crude means and standard deviations. [file 12889_2024_18954_MOESM4_ESM.pdf]

## APPENDIX D: CRUDE MEANS AND STANDARD DEVIATIONS

**Table D1. Descriptive statistics of physical activity & sedentary behaviour pre-lockdown and during lockdown – Mean (SD)**

| | T-2 | T-1 | T0 | T1 | T2 |
|---|---|---|---|---|---|
| | **Mean (SD) (min/week)** | **Mean (SD) (min/week)** | **Mean (SD) (min/week)** | **Mean (SD) (min/week)** | **Mean (SD) (min/week)** |
| **Total PA** | 1771·16 (1367·43) | 1405·88 (1165·88) | 1376·51 (1156·12) | 1436·99 (1114·21) | 1322·05 (1064·46) |
| Walking | 626·46 (743·19) | 495·98 (612·86) | 499·13 (648·01) | 406·44 (457·29) | 337·04 (399·20) |
| Moderate-intensity PA | 1001·62 (885·02) | 811·59 (738·31) | 769·49 (696·74) | 936·65 (855·72) | 878·12 (822·97) |
| Vigorous-intensity PA | 143·08 (297·51) | 98·31 (208·97) | 107·89 (246·19) | 93·91 (220·13) | 106·89 (223·00) |
| Work-related PA | 654·70 (838·02) | 516·34 (685·06) | 520·05 (710·65) | 209·18 (469·88) | 196·34 (406·80) |
| Transport-related PA | 255·21 (321·75) | 214·64 (266·18) | 225·27 (337·72) | 180·38 (259·61) | 194·49 (249·80) |
| Domestic and garden PA | 597·30 (647·46) | 473·85 (561·47) | 418·07 (452·73) | 719·74 (702·64) | 593·97 (674·93) |
| Leisure-time PA | 263·95 (383·50) | 201·05 (312·08) | 213·12 (364·95) | 327·69 (382·17) | 337·25 (400·70) |
| **Total SB** | 3160·47 (1121·34) | 3088·30 (1129·64) | 3135·04 (1083·21) | 4098·01 (1234·93) | 4013·63 (1194·96) |
| Work-related SB | 755·43 (462·17) | 710·14 (536·45) | 671·35 (460·14) | 1333·11 (859·18) | 1545·59 (926·24) |
| Transport-related SB | 419·53 (351·61) | 398·94 (338·70) | 398·15 (332·38) | 107·87 (267·39) | 172·34 (248·82) |
| Leisure-time SB | 1985·52 (914·49) | 1979·21 (909·34) | 2065·54 (897·95) | 2657·03 (1044·91) | 2295·70 (962·05) |

*Abbreviations: SD = standard deviation; PA = physical activity; SB = sedentary behaviour*
